# Supplementary material for: Abundance Redistribution Increases Predator‐Prey Interaction Potentials Among North American Birds
Source: Glob Chang Biol. 2026 Nov 3;32(11):e71163. doi: 10.1111/gcb.71163 (PMC13731941; doi:10.1111/gcb.71163)
Supplement: Supplementary file 1 [file GCB-32-e71163-s001.pdf]

Supplementary Material for

Abundance redistribution increases predator-prey interaction potentials among North American birds

Heng-Xing Zou, Chia Hsieh, Phoebe L. Zarnetske, Brian C. Weeks, and Kai Zhu

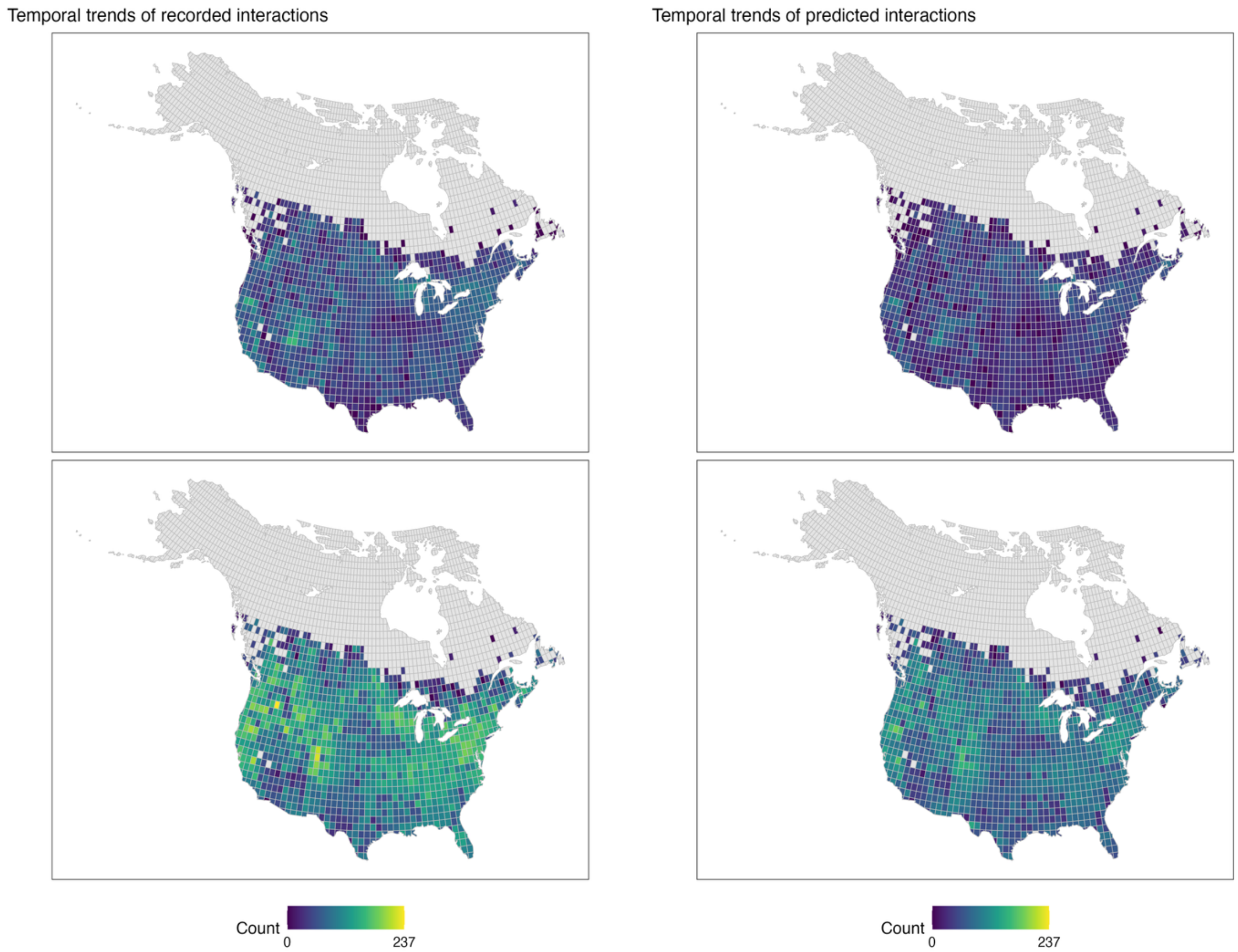

Figure S1. Map of temporal trends of interaction potentials in each local community. Top row: number of predator-prey interactions with decreasing interaction potential; bottom row: number of predator-prey interactions with increasing interaction potential. Left column: interactions recorded in the existing dataset; right column: interactions predicted from the existing dataset. Only species pairs with statistically significant temporal trends of interaction potentials are included. Numbers are sums of species pairs across all local communities: if a species pair shows significantly increasing interaction potential in *n* local communities, it will be counted *n* times.

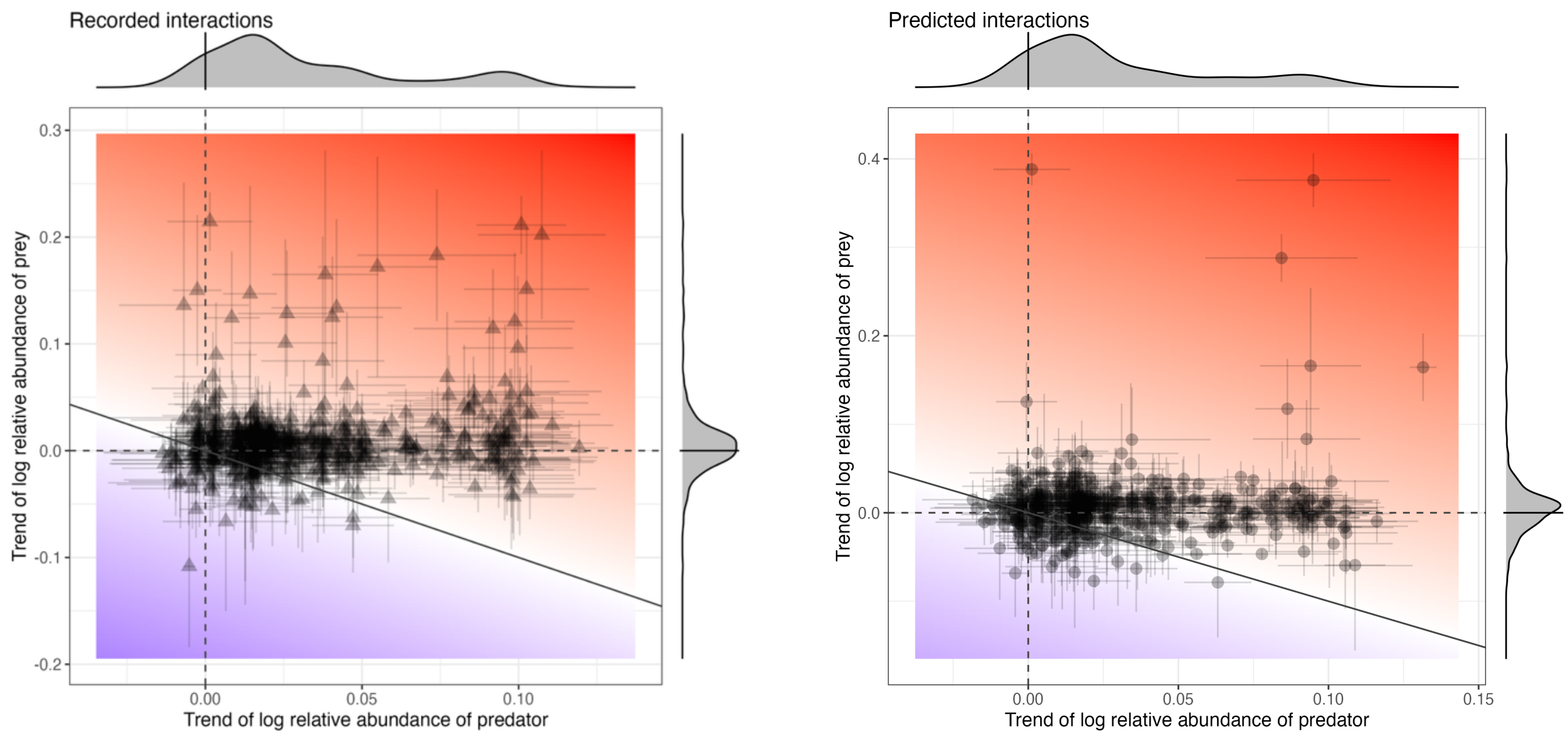

Figure S2. Contribution of predators and prey to temporal changes in their interaction potentials, for all predator-prey pairs and in all local communities. The x-axis represents the temporal trend of predator relative abundances, and the y-axis represents the temporal trend of prey relative abundances. Pairs above the 1:1 line indicate increasing interaction potentials over time (i.e., positive temporal trends). Marginal density plots represent the distribution of temporal trends. Panel A (left): interactions recorded in the existing dataset; panel B (right): interactions predicted from the existing dataset. Only species pairs with statistically significant temporal trends of interaction potentials are included.

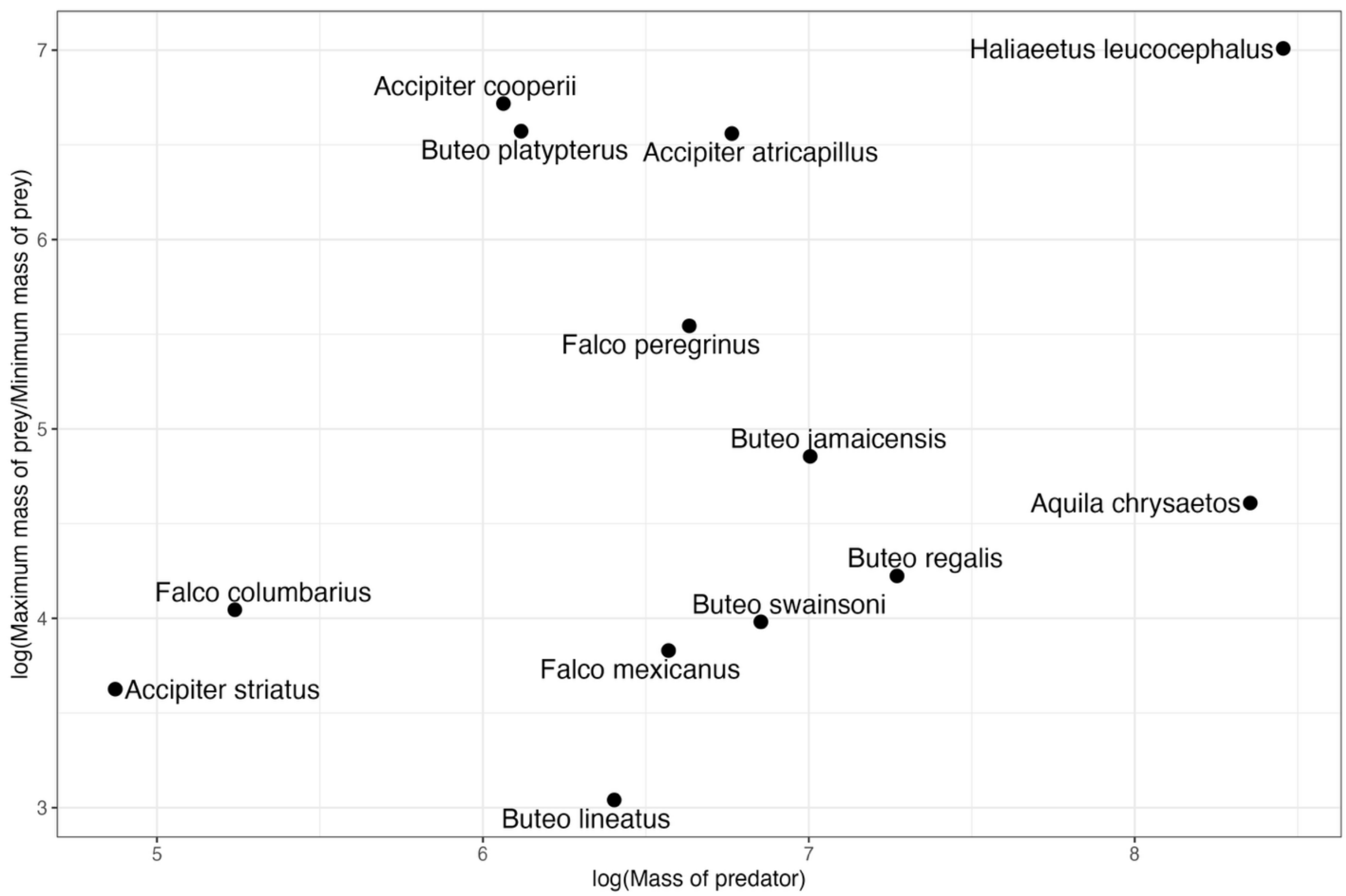

Figure S3. Ratio of maximum to minimum body mass, log-transformed, of recorded prey species for 14 predators included in the study. Larger ratios indicate that the predator species can prey on species with wider ranges of body mass.

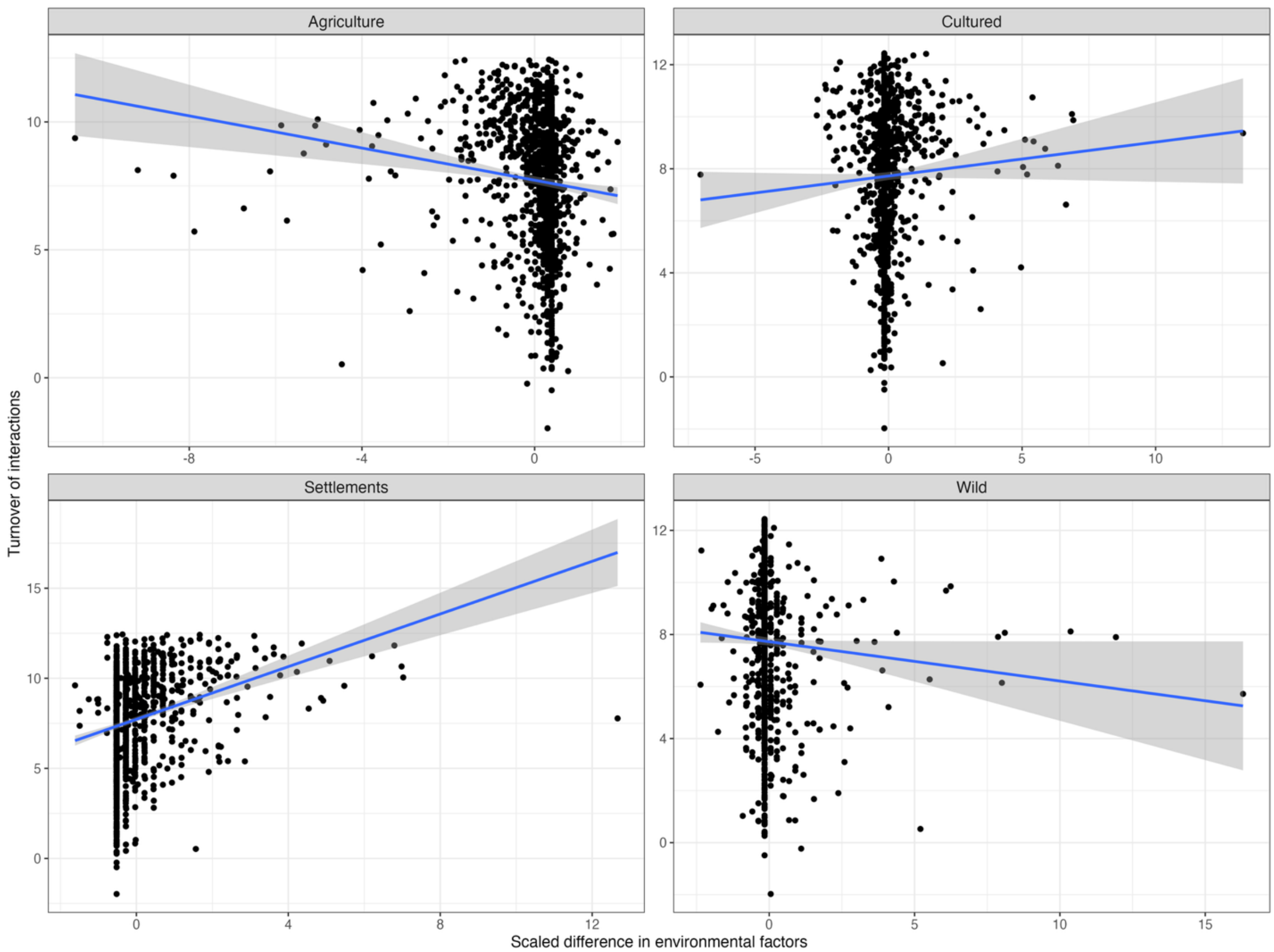

Figure S4. Associations between turnover of interaction networks in local communities (log-transformed; y-axis) and differences in four categories of human-induced land use changes (x-axis). The association with changes in wild land is the weakest.

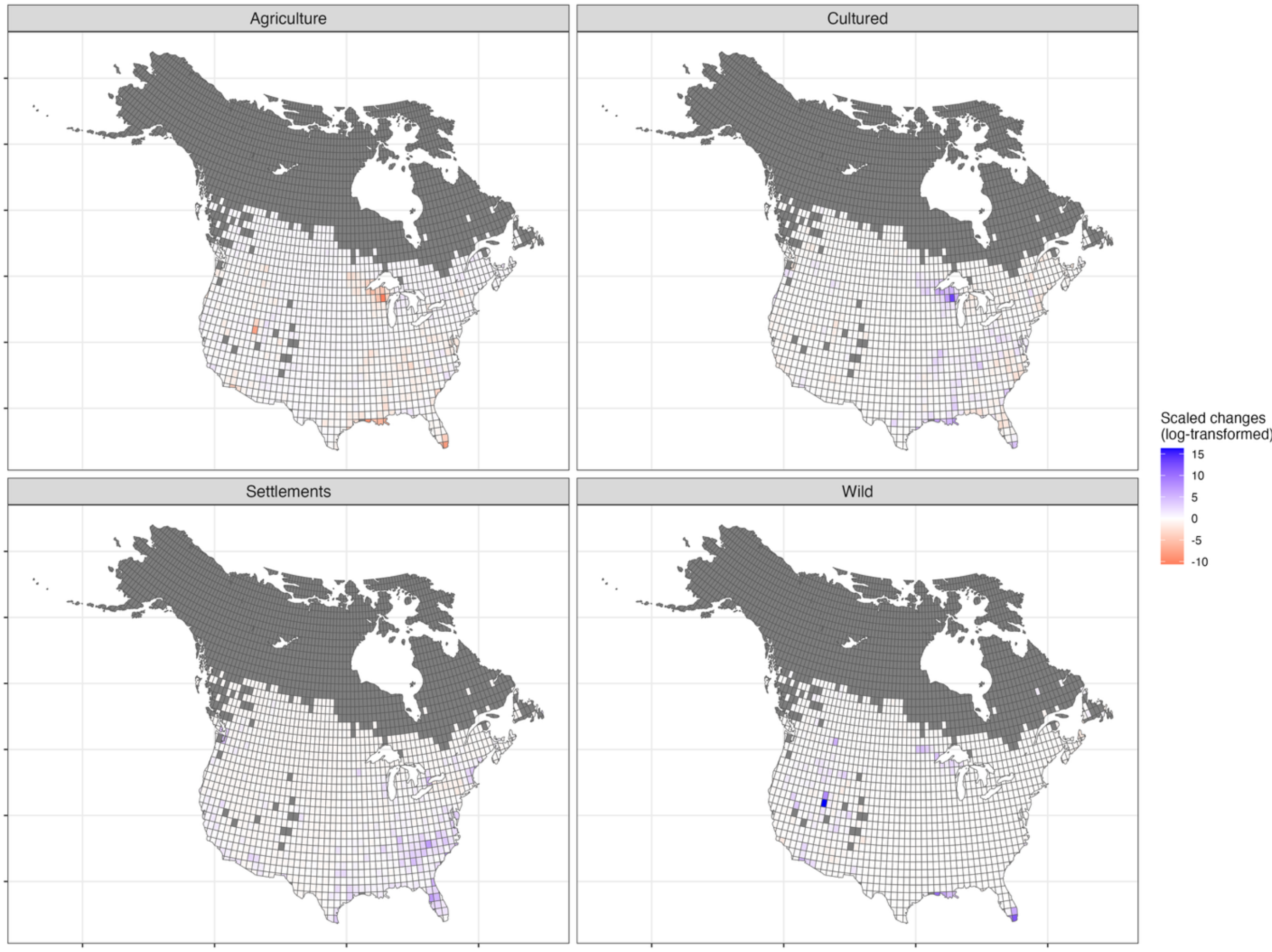

Figure S5. Maps of changes in four human-induced land use types between 1970 and 2021 in each one-degree latitude and longitude grid; see **Methods** for details of the calculations. These maps show the spatial variation in land use changes for different land types.

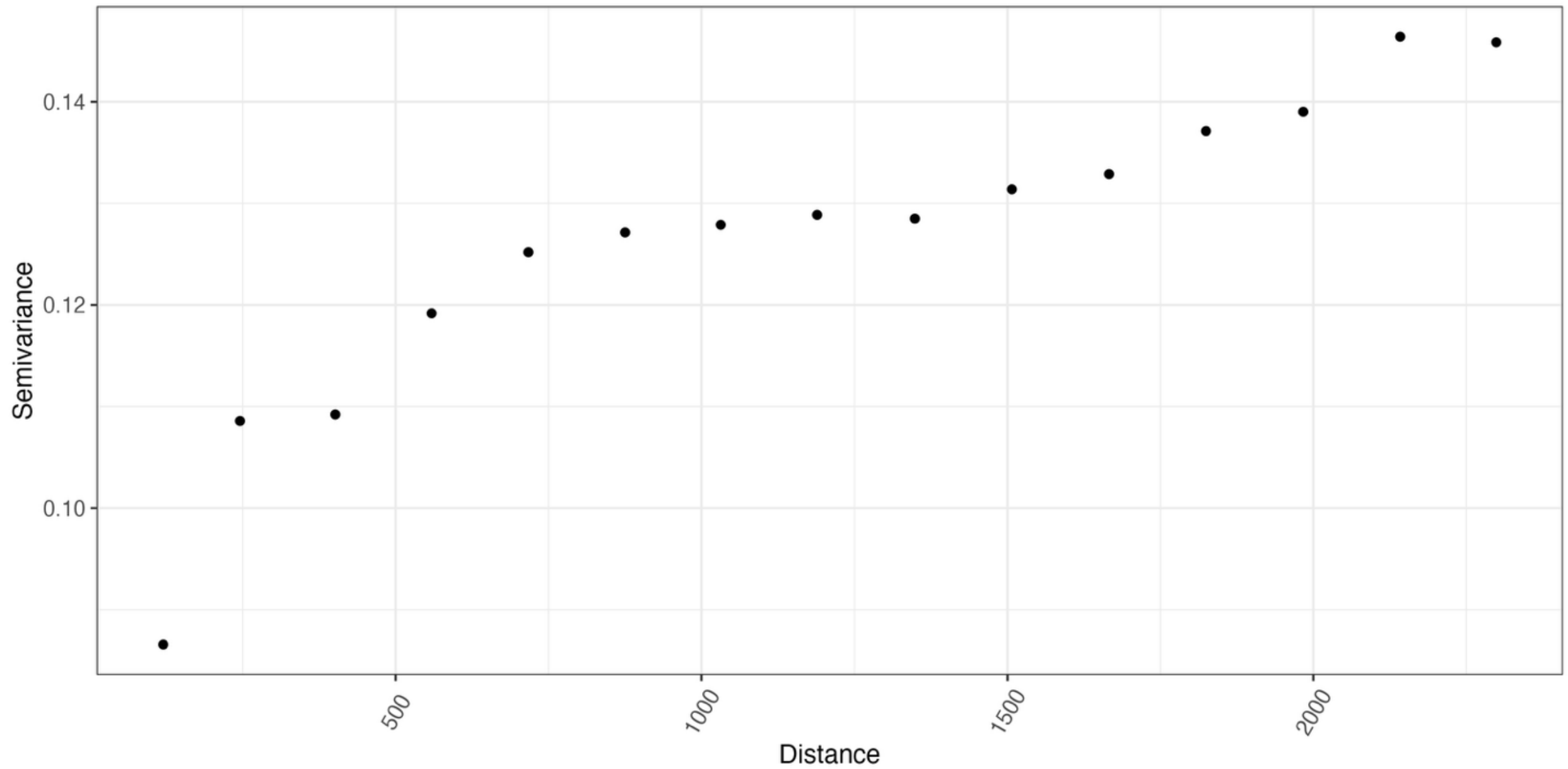

Figure S6. The variogram of the non-spatial linear model on turnover of interaction potentials between 1970 and 2021. See *Methods* for details of the model. The x-axis represents the distance, and the y-axis indicates the semivariance in the residuals of the model. The semivariance increases almost monotonically over distance, indicating a strong spatial signal in the residual of the non-spatial linear model that shows no sign of tapering off at a range of 2000km.

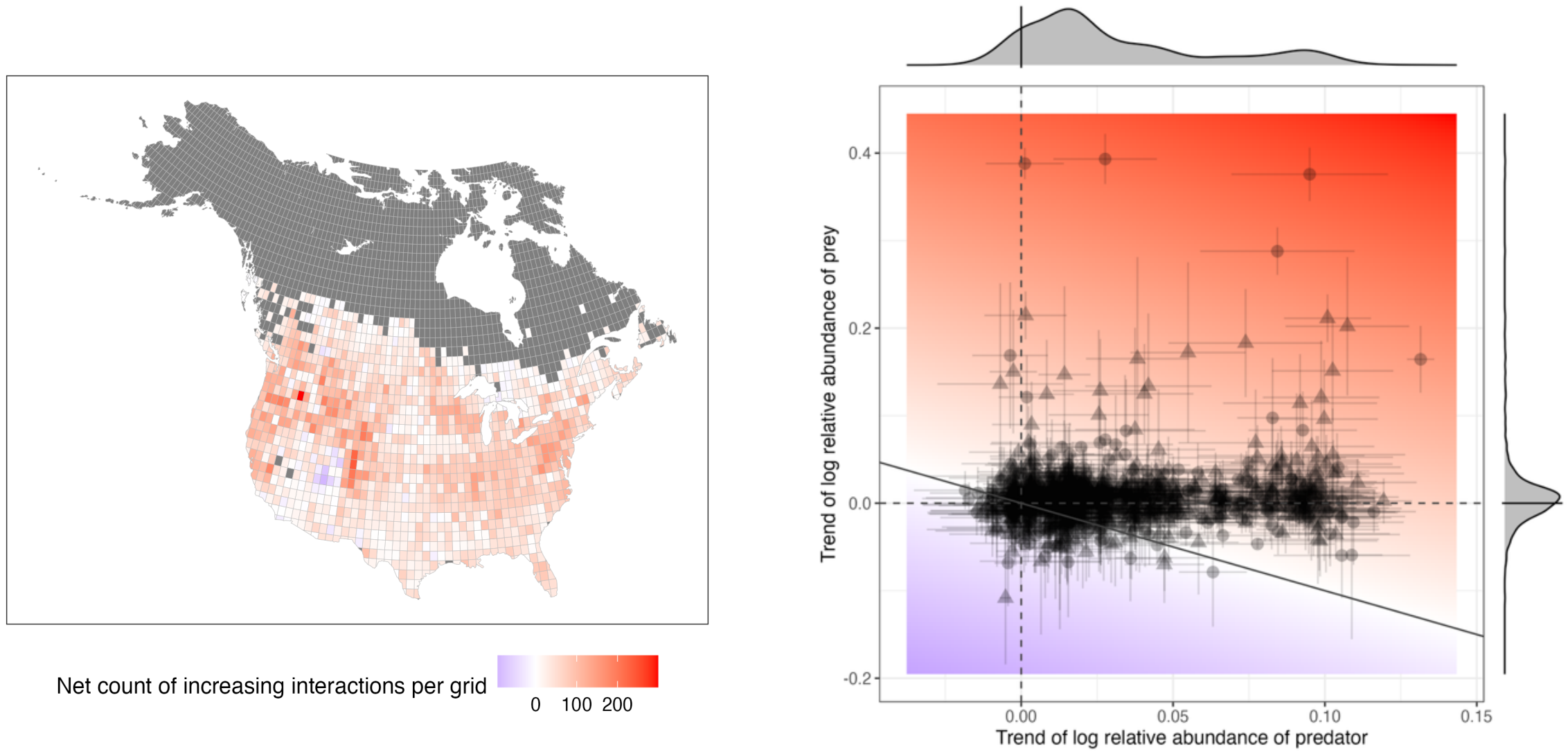

Figure S7. Most pairwise predator-prey interactions have increased their potential over time due to the increasing relative abundance of predators, not prey. These results are part of the sensitivity analysis, where we allowed for the threshold of functional compatibility to scale with the number of species in a community. Panel A (left): map showing the net count of increased interaction potentials, calculated by the number of pairs with increasing interaction potentials minus that of decreasing potentials in each one-degree latitude and longitude grid. Panel B (right): contribution of predators and prey to temporal changes in their interaction potentials, for all predator-prey pairs and in all local communities. For each pairwise interaction, the x-axis represents the mean temporal trend of predator relative abundances, and the y-axis represents the mean temporal trend of prey relative abundances, with associated standard deviations. Pairs above the 1:1 line indicate increasing interaction potentials over time (i.e., positive temporal trends). Marginal density plots represent the distribution of temporal trends. Both panels included interactions recorded in the existing dataset (triangles), and interactions predicted by the "functional compatibility" (dots). For both panels, one species pair will be counted multiple times if its interaction potential significantly increases/decreases in multiple local communities. Compare to Figure 1 where functional compatibility does not scale with the number of species in a community.

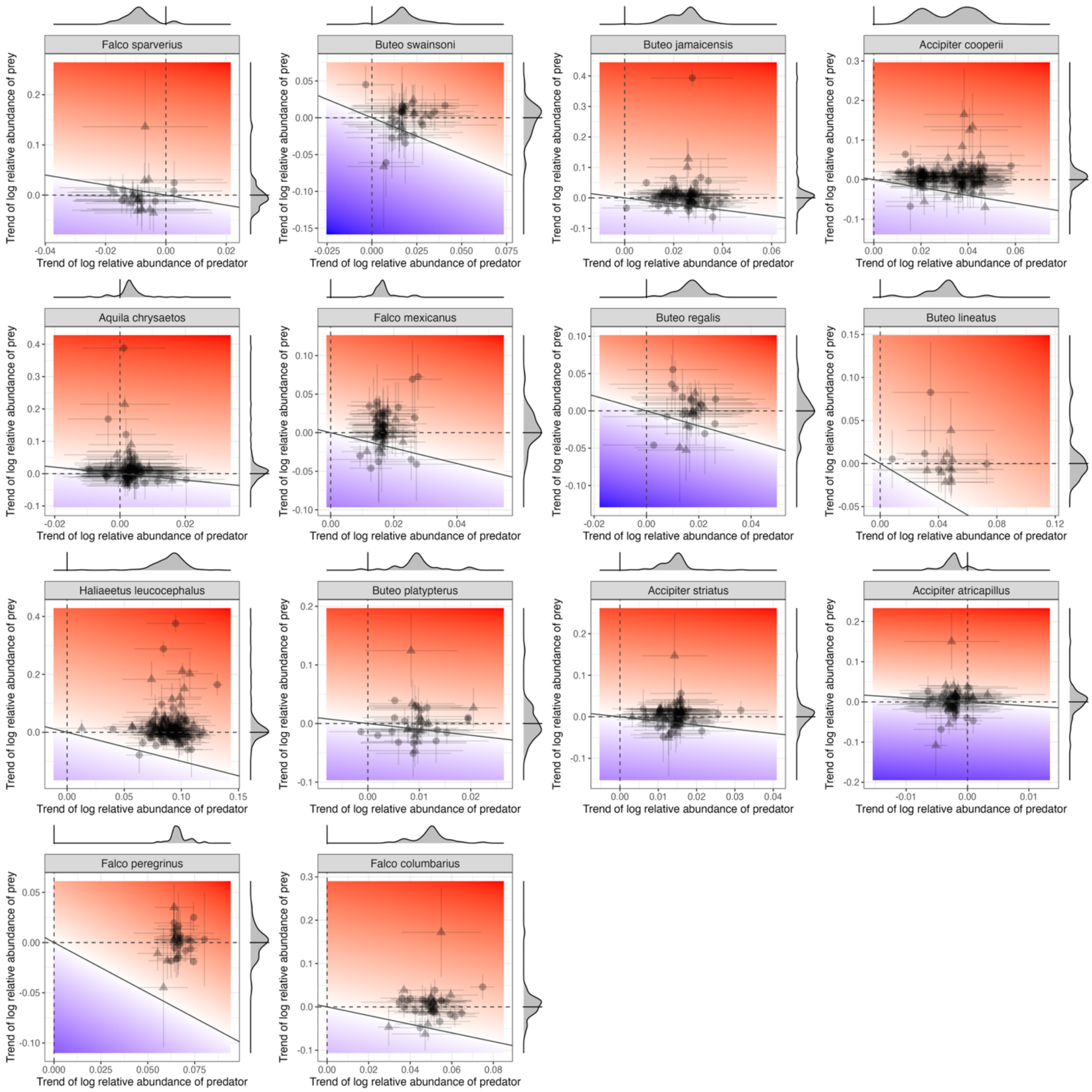
Falco sparverius
Buteo swainsoni
Buteo jamaicensis
Accipiter cooperii
Aquila chrysaetos
Falco mexicanus
Buteo regalis
Buteo lineatus
Haliaeetus leucocephalus
Buteo platypterus
Accipiter striatus
Accipiter atricapillus
Falco peregrinus
Falco columbarius
Trend of log relative abundance of predator
Trend of log relative abundance of prey

Figure S8. Contribution of predators and prey to temporal changes in their interaction potentials, for all pairs across all local communities by predator species. These results are part of the sensitivity analysis, where we allowed for the threshold of functional compatibility to scale with the number of species in a community. For each pairwise interaction, the x-axis represents the mean temporal trend of predator relative abundances, and the y-axis represents the mean temporal trend of prey relative abundances, with associated standard deviations. Pairs above the 1:1 line indicate increasing interaction potentials over time (i.e., positive temporal trends). Marginal density plots represent the distribution of temporal trends. All panels included interactions recorded in the existing dataset (triangles), and interactions predicted by the “functional compatibility” (dots). One species pair will be counted multiple times if its interaction potential significantly increases/decreases in multiple local communities. Compare to Figure 2 where functional compatibility does not scale with the number of species in a community.

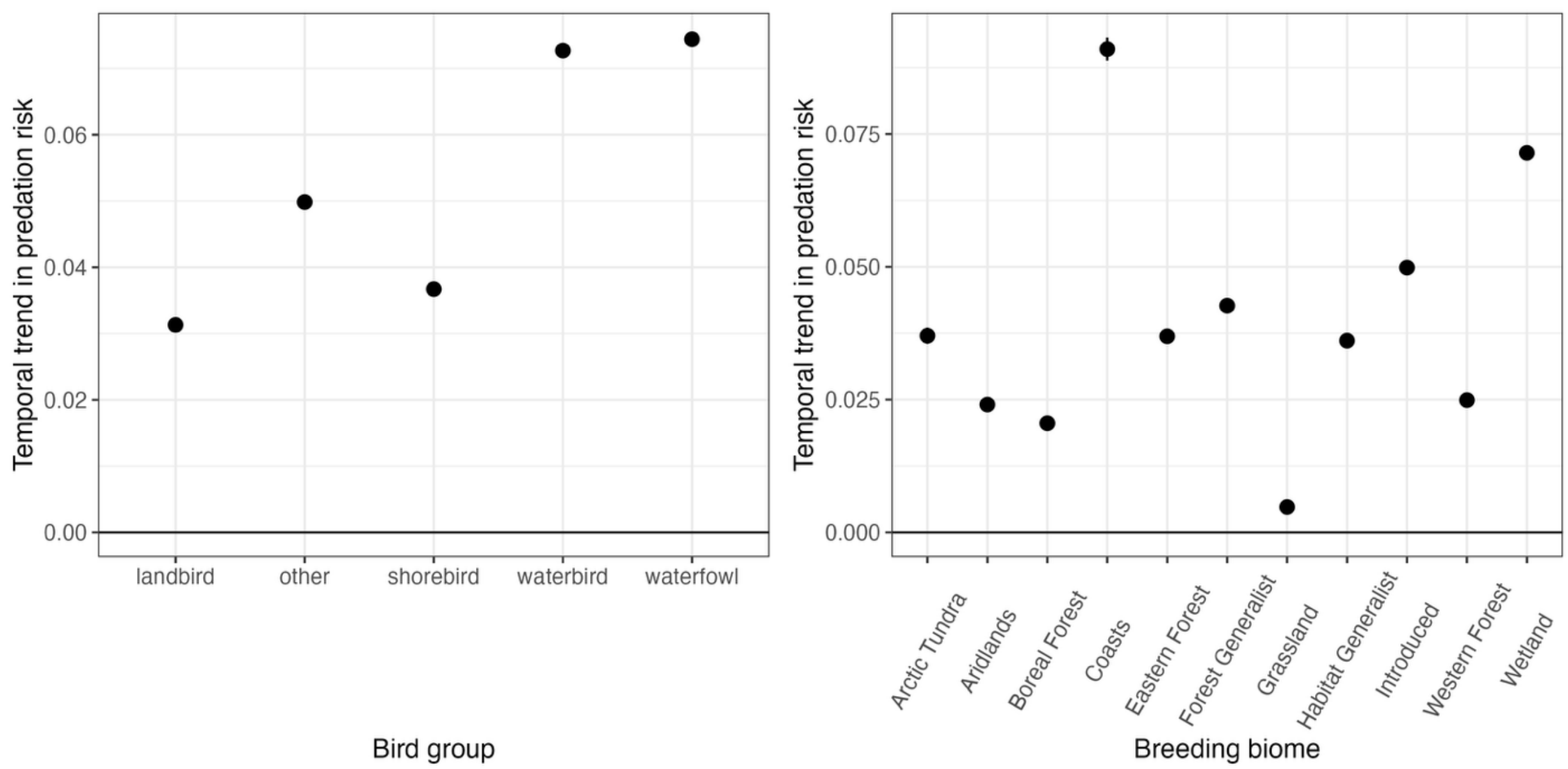

Figure S9. Temporal trends of overall predation risks by functional groups and breeding biomes of recorded and predicted prey species; points and lines indicate effect sizes and standard errors. These results are part of the sensitivity analysis, where we allowed for the threshold of functional compatibility to scale with the number of species in a community. Panel A (left): temporal trends by functional group; panel B (right): temporal trends by breeding biomes. Compare to Figure 3 where functional compatibility does not scale with the number of species in a community.

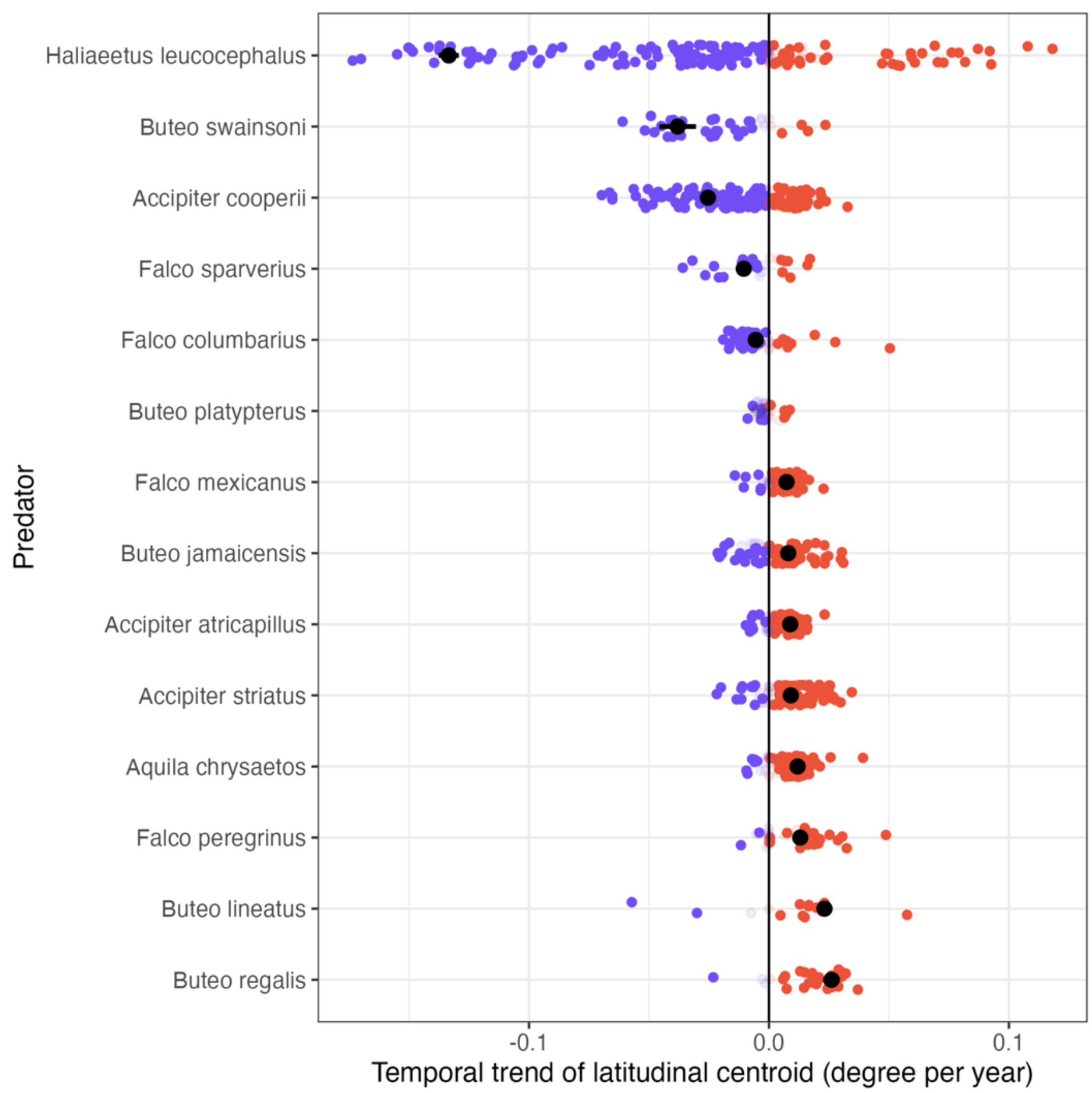

Figure S10. Latitudinal shifts over time of predator abundances in their entire ranges (black dots), and in their spatial overlap with their prey species (colored dots), including both recorded and predicted interactions. These results are part of the sensitivity analysis, where we allowed for the threshold of functional compatibility to scale with the number of species in a community. For a predator, because the spatial overlap with each prey species is different, each predator-prey pair will have an individual latitudinal shift. On the x-axis, positive numbers indicate northward shifts, negative numbers indicate southward shifts. Non-significant shifts are shown in lighter shades. Compare to Figure 4 where functional compatibility does not scale with the number of species in a community.

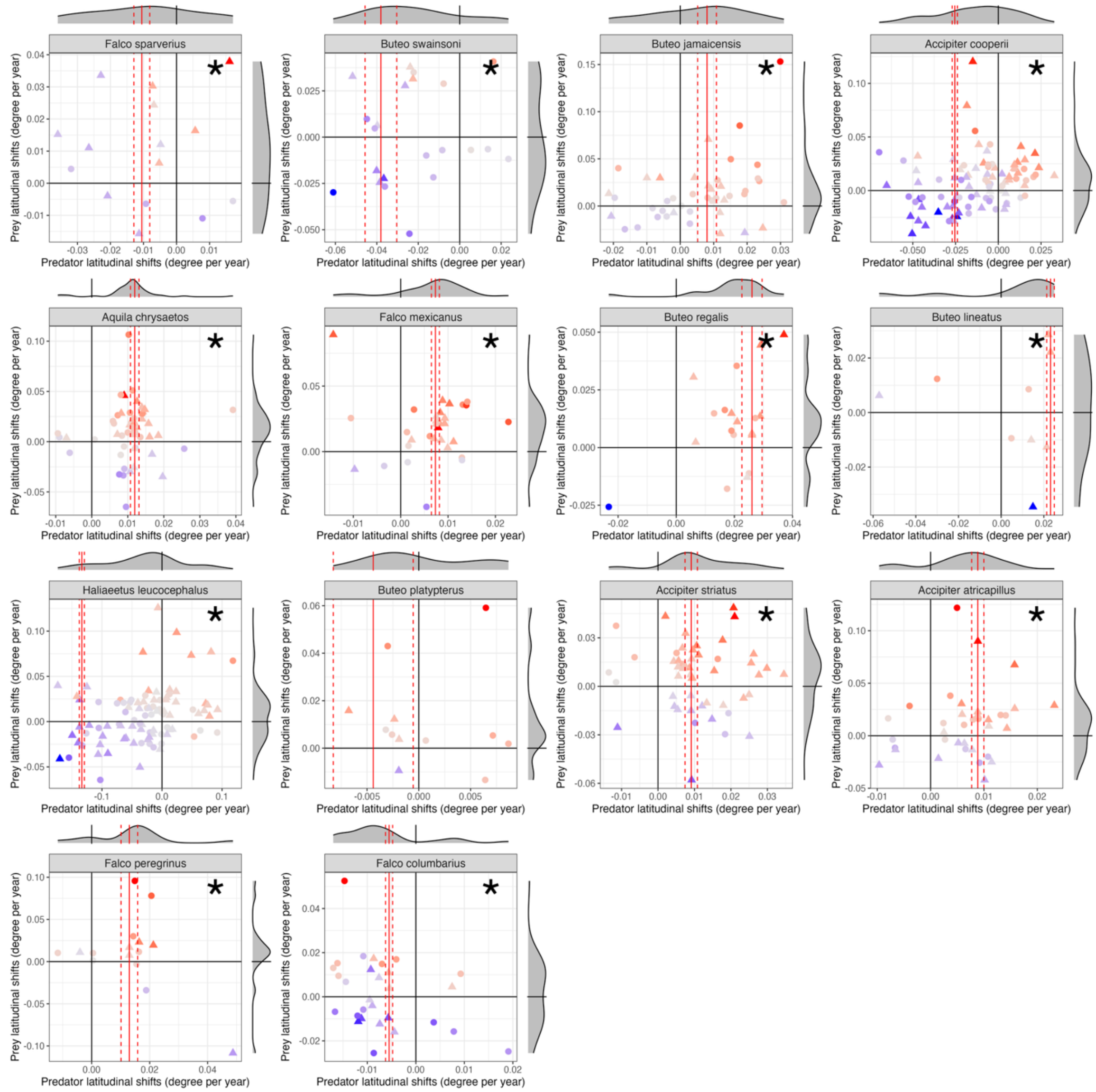

Figure S11. Latitudinal shifts of interaction potentials, decomposed by the latitudinal shifts of predators and prey abundances. Each panel represents both recorded and predicted interactions of one predator. These results are part of the sensitivity analysis, where we allowed for the threshold of functional compatibility to scale with the number of species in a community. The x- and y-axes indicate the latitudinal shifts of predators and prey, respectively, with positive values indicating northward shifts, and the marginal density plots show the distribution of latitudinal shifts, with black solid lines indicating 0 (no shift). Colors of each dot indicate the latitudinal shifts of each pairwise interaction potential (red: shifting significantly north; blue: shifting significantly south), dots indicate predicted interactions, and triangles indicate recorded interactions. The red vertical lines indicate the fitted slope and standard errors of the overall latitudinal shift of the predator, and the asterisks indicate that the means of the distributions of shifts in predator interaction potentials are significantly different from zero. Compare to Figure 5 where functional compatibility does not scale with the number of species in a community.

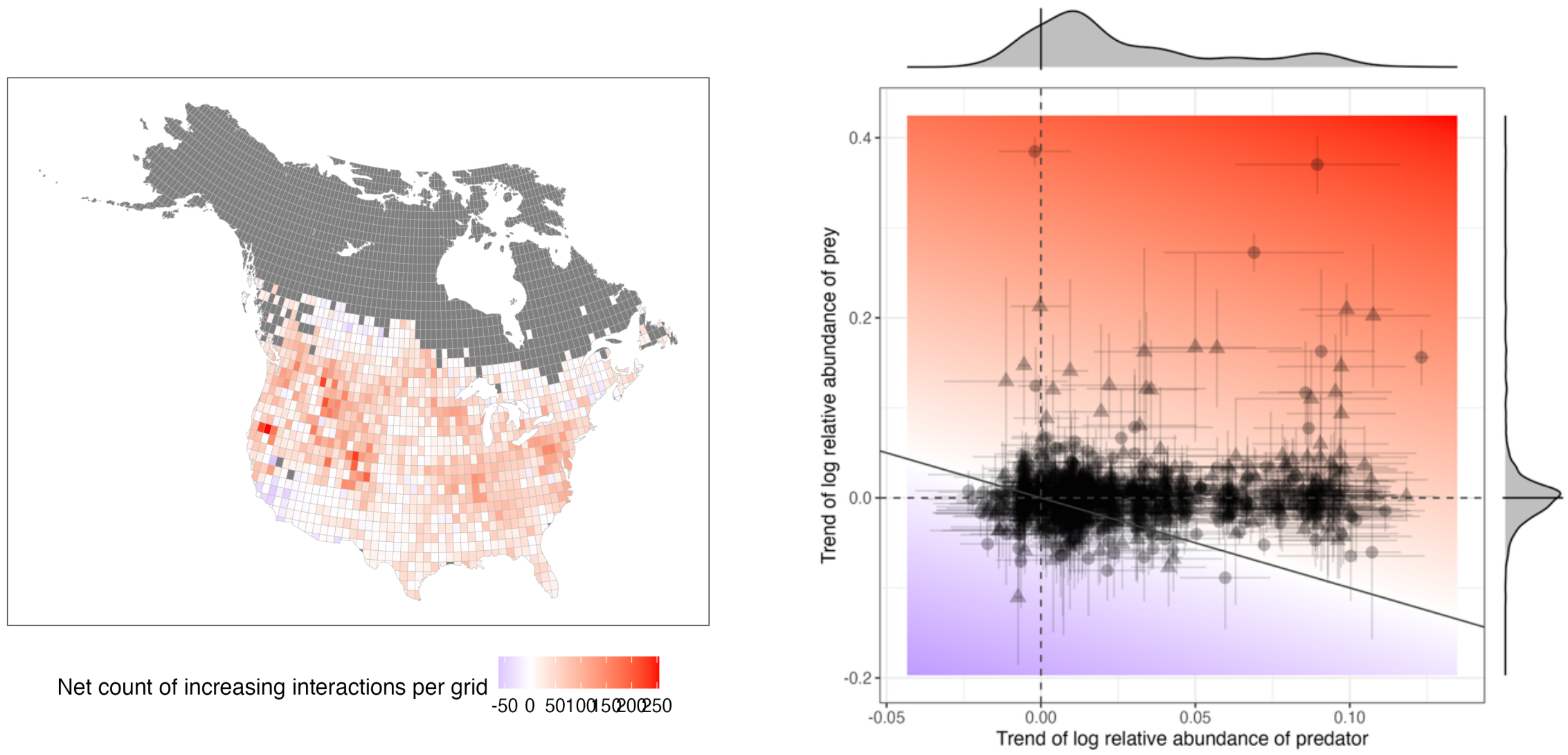

Figure S12. Most pairwise predator-prey interactions have increased their potential over time due to the increasing absolute abundance of predators, not prey. These results are part of the sensitivity analysis, where we calculated interaction potentials using absolute instead of relative abundances. Panel A (left): map showing the net count of increased interaction potentials, calculated by the number of pairs with increasing interaction potentials minus that of decreasing potentials in each one-degree latitude and longitude grid. Panel B (right): contribution of predators and prey to temporal changes in their interaction potentials, for all predator-prey pairs and in all local communities. For each pairwise interaction, the x-axis represents the mean temporal trend of predator relative abundances, and the y-axis represents the mean temporal trend of prey relative abundances, with associated standard deviations. Pairs above the 1:1 line indicate increasing interaction potentials over time (i.e., positive temporal trends). Marginal density plots represent the distribution of temporal trends. Both panels included interactions recorded in the existing dataset (triangles), and interactions predicted by the "functional compatibility" (dots). For both panels, one species pair will be counted multiple times if its interaction potential significantly increases/decreases in multiple local communities. Compare to Figure 1's results with relative abundances.

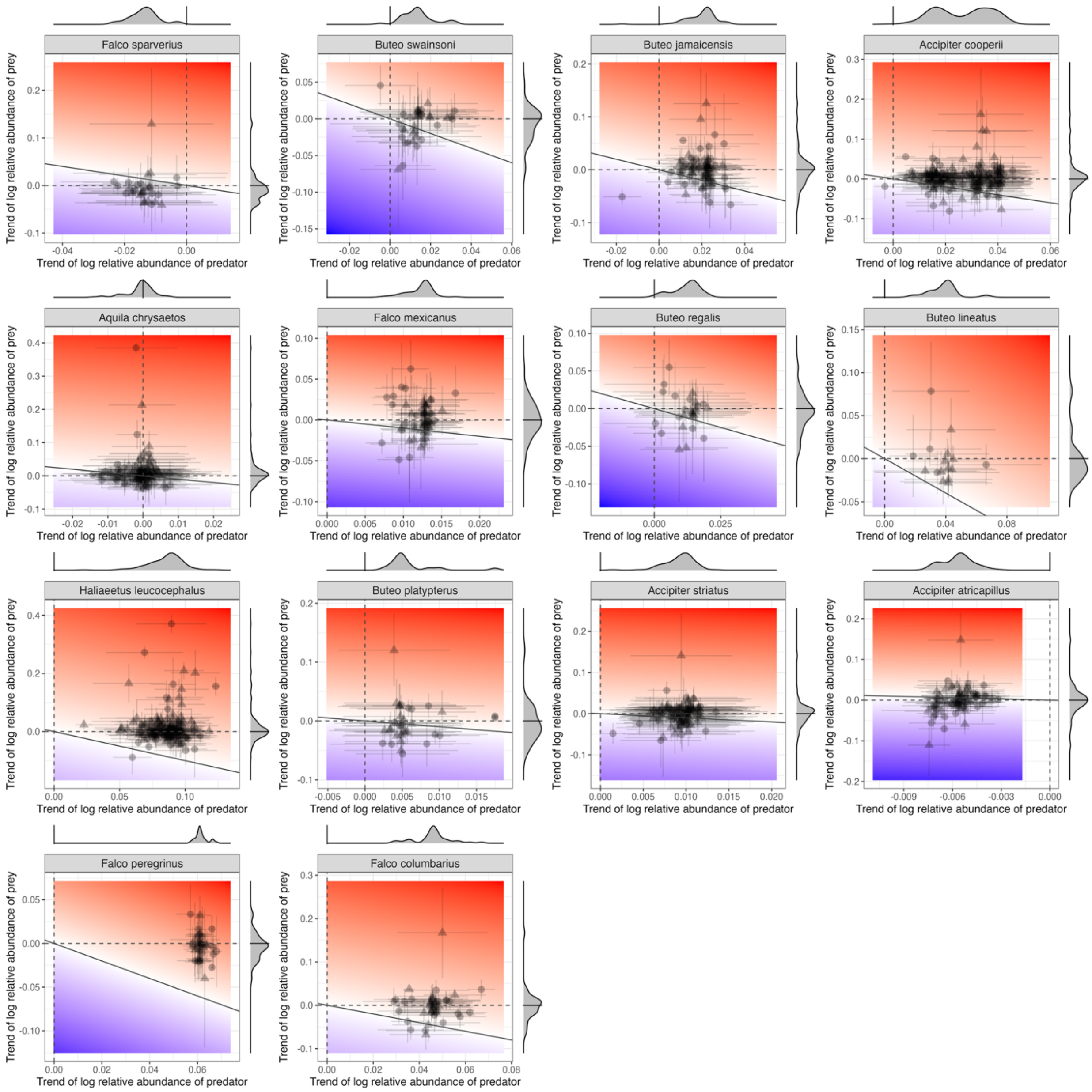

Figure S13. Contribution of predators and prey to temporal changes in their interaction potentials, for all pairs across all local communities by predator species. These results are part of the sensitivity analysis, where we calculated interaction potentials using absolute instead of relative abundances. For each pairwise interaction, the x-axis represents the mean temporal trend of predator relative abundances, and the y-axis represents the mean temporal trend of prey relative abundances, with associated standard deviations. Pairs above the 1:1 line indicate increasing interaction potentials over time (i.e., positive temporal trends). Marginal density plots represent the distribution of temporal trends. All panels included interactions recorded in the existing dataset (triangles), and interactions predicted by the "functional compatibility" (dots). One species pair will be counted multiple times if its interaction potential significantly increases/decreases in multiple local communities. Compare to Figure 2's results with relative abundances

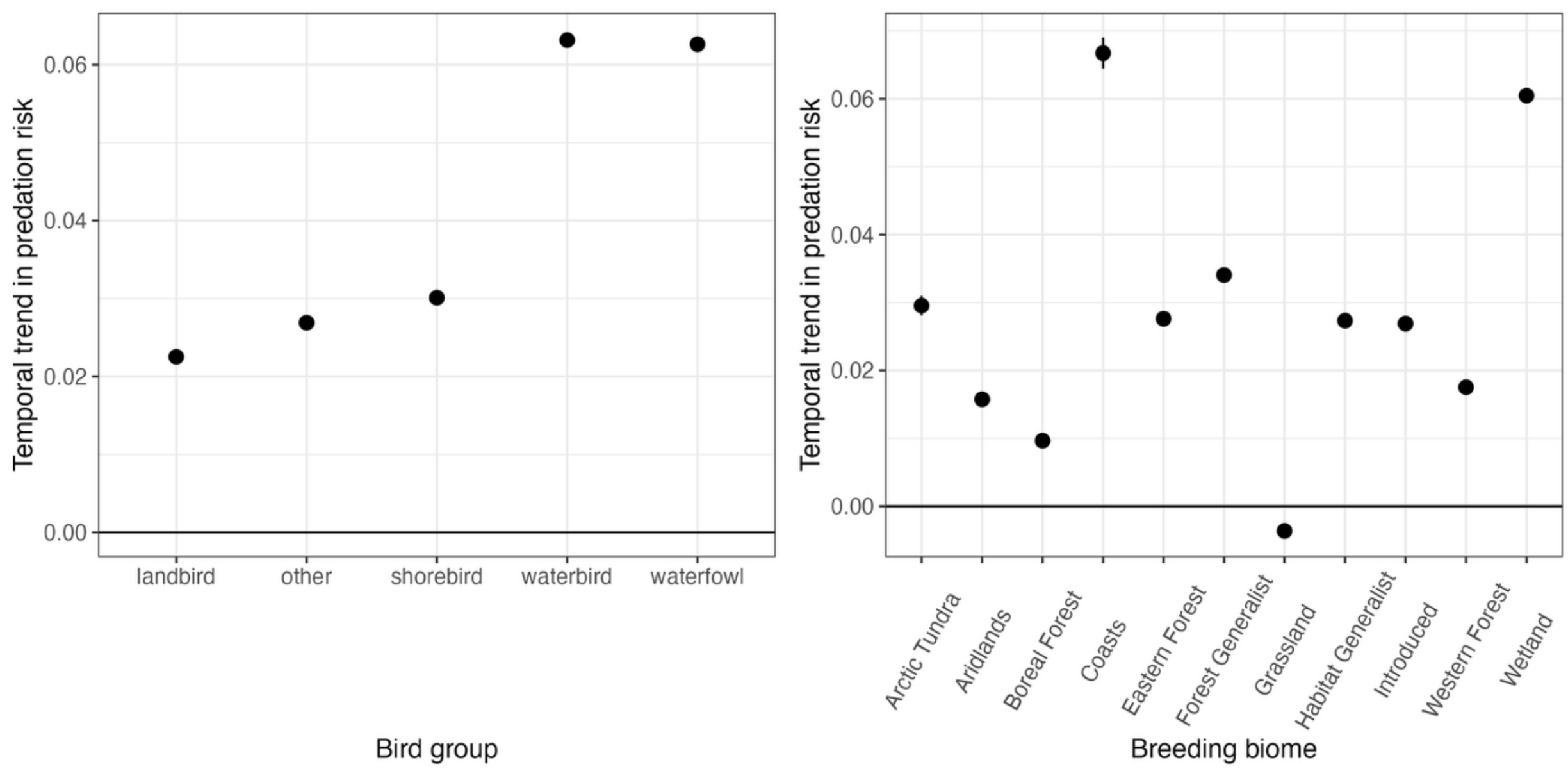

Figure S14. Temporal trends of overall predation risks by functional groups and breeding biomes of recorded and predicted prey species; points and lines indicate effect sizes and standard errors. These results are part of the sensitivity analysis, where we calculated interaction potentials using absolute instead of relative abundances. Panel A (left): temporal trends by functional group; panel B (right): temporal trends by breeding biomes. Note that grassland birds show decreasing predation risks over time when calculated using absolute abundances, possibly due to the strong decline in their populations over time. Compare to Figure 3’s results with relative abundances

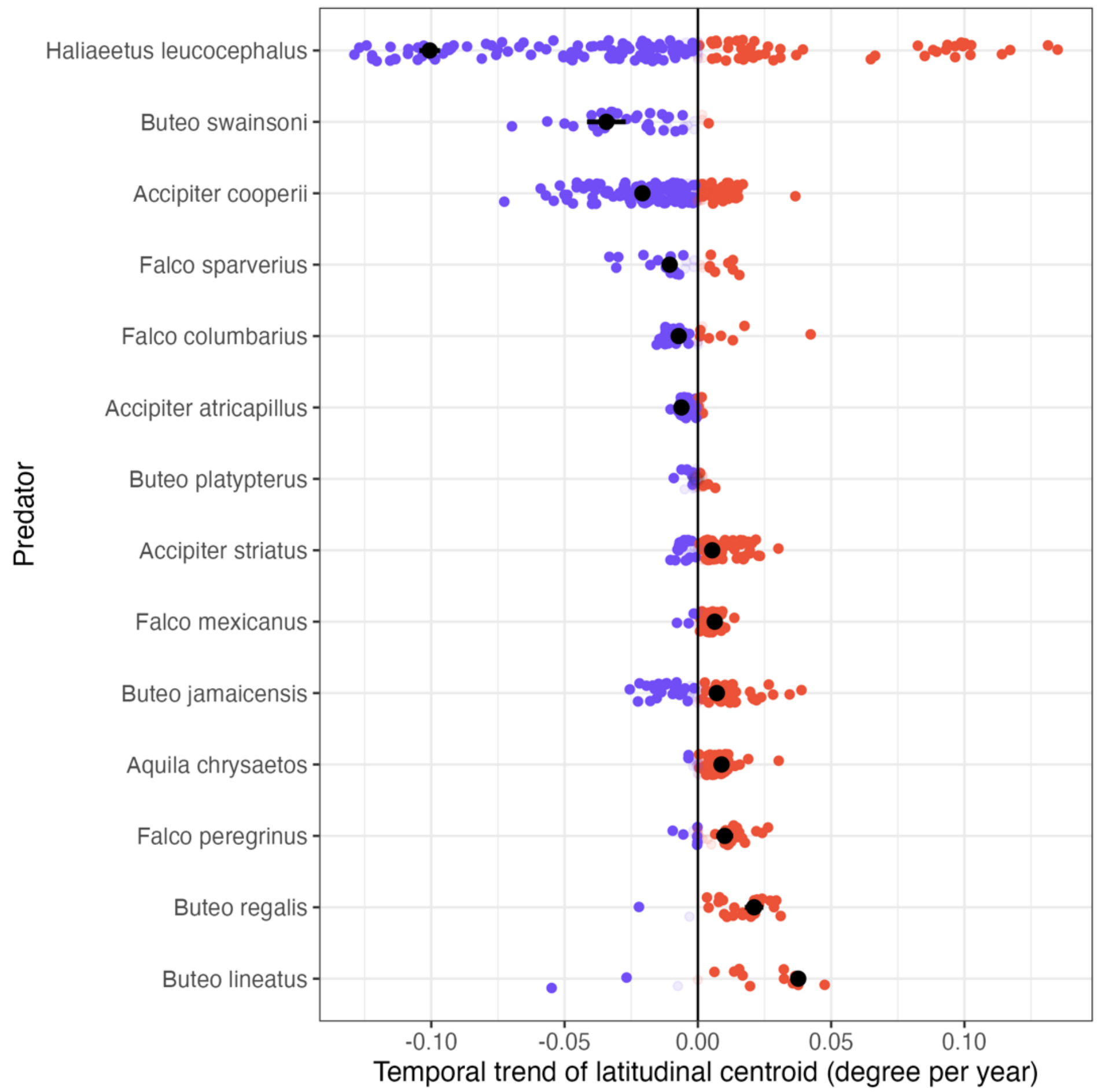

Figure S15. Latitudinal shifts over time of predator abundances in their entire ranges (black dots), and in their spatial overlap with their prey species (colored dots), including both recorded and predicted interactions. These results are part of the sensitivity analysis, where we calculated interaction potentials using absolute instead of relative abundances. For a predator, because the spatial overlap with each prey species is different, each predator-prey pair will have an individual latitudinal shift. On the x-axis, positive numbers indicate northward shifts, negative numbers indicate southward shifts. Non-significant shifts are shown in lighter shades. Compare to Figure 4's results with relative abundances

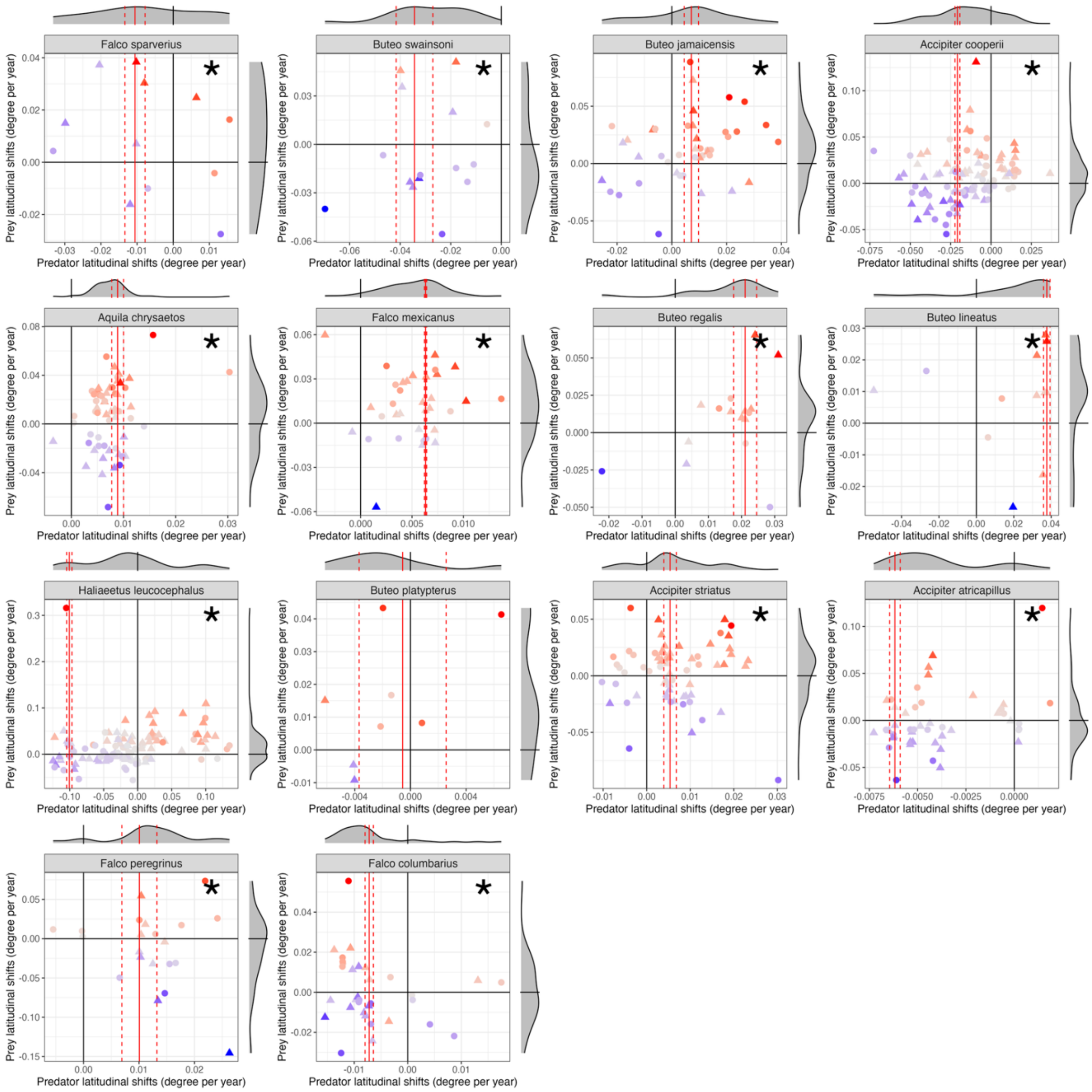

Figure S16. Latitudinal shifts of interaction potentials, decomposed by the latitudinal shifts of predators and prey abundances. Each panel represents both recorded and predicted interactions of one predator. These results are part of the sensitivity analysis, where we calculated interaction potentials using absolute instead of relative abundances. The x- and y-axes indicate the latitudinal shifts of predators and prey, respectively, with positive values indicating northward shifts, and the marginal density plots show the distribution of latitudinal shifts, with black solid lines indicating 0 (no shift). Colors of each dot indicate the latitudinal shifts of each pairwise interaction potential (red: shifting significantly north; blue: shifting significantly south), dots indicate predicted interactions, and triangles indicate recorded interactions. The red vertical lines indicate the fitted slope and standard errors of the overall latitudinal shift of the predator, and the asterisks indicate that the means of the distributions of shifts in predator interaction potentials are significantly different from zero. Compare to Figure 5's results with relative abundances.

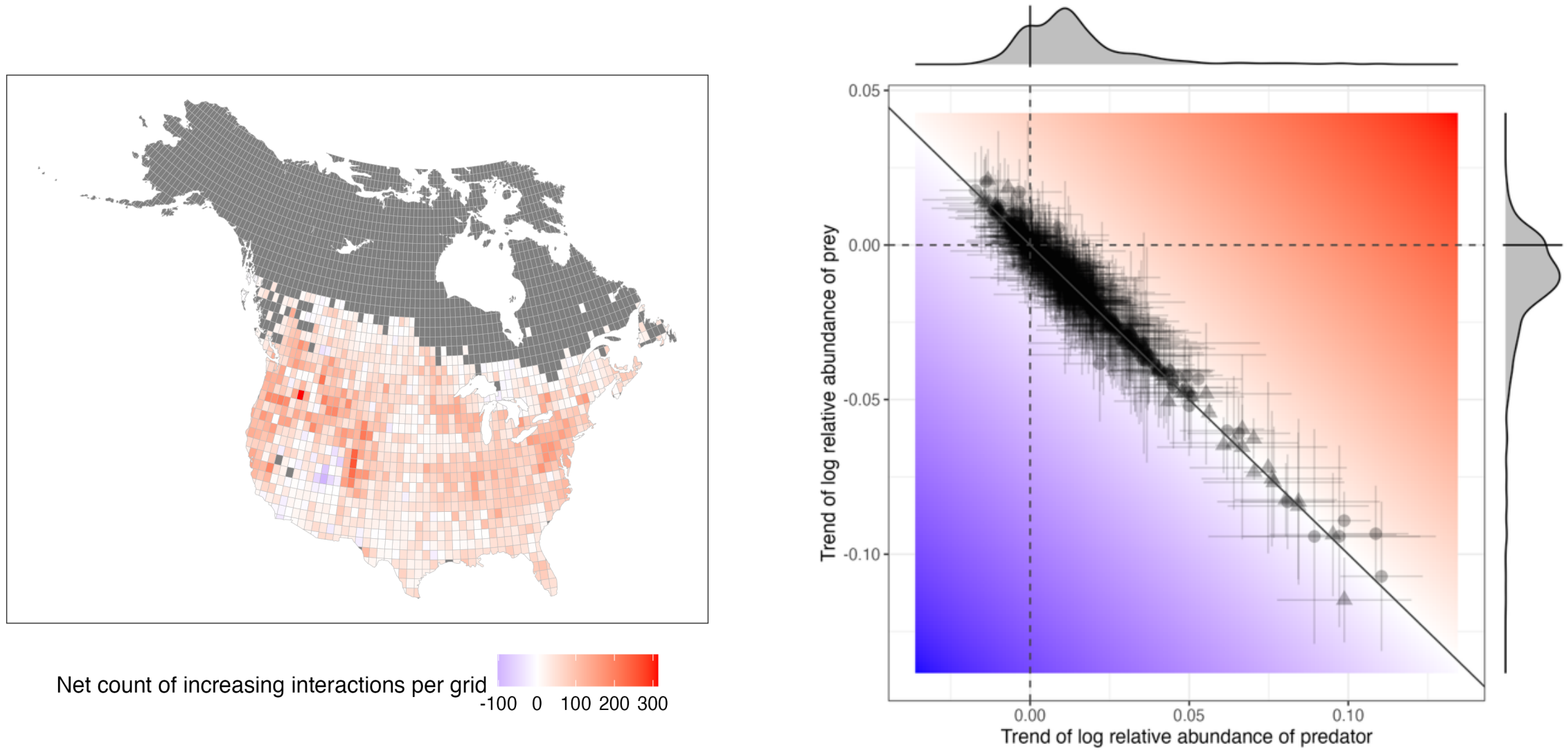

Figure S17. Most pairwise predator-prey interactions have increased their potential over time due to the increasing abundance of predators, not prey. Panel A (left): map showing the net count of increased interaction potentials, calculated by the number of pairs with increasing interaction potentials minus that of decreasing potentials in each one-degree latitude and longitude grid, including pairs that show both significant and non-significant temporal trends. Panel B (right): contribution of predators and prey to temporal changes in their interaction potentials, for predator-prey pairs showing non-significant temporal trends only. For each pairwise interaction, the x-axis represents the mean temporal trend of predator relative abundances, and the y-axis represents the mean temporal trend of prey relative abundances, with associated standard deviations. Pairs above the 1:1 line indicate increasing interaction potentials over time (i.e., positive temporal trends). Marginal density plots represent the distribution of temporal trends. Both panels included interactions recorded in the existing dataset (triangles) and interactions predicted by the "functional compatibility" (dots). For both panels, one species pair will be counted multiple times if its interaction potential significantly increases/decreases in multiple local communities. Compare to Figure 1's results with interaction pairs with significant temporal trends only.

Table S1. Statistics of linear mixed effects models for the temporal trends of predation risks for prey species by groups. The upper table shows the results by functional groups, lower table shows the results by breeding biomes.

Parameters	Estimate	Std. Error	df	t value	Adjusted *p* value	Group
(Intercept)	-15.811829	0.1319793	271.197206	-119.80537	**<0.00001**	landbird
year_since_1969	0.03147079	8.24E-05	2773348.23	382.121203	**<0.00001**	landbird
(Intercept)	-19.49082	0.37330267	12.679676	-52.211841	**<0.00001**	shorebird
year_since_1969	0.03815552	3.35E-04	92543.4978	113.990421	**<0.00001**	shorebird
(Intercept)	-21.475931	0.2459613	44.991039	-87.31427	**<0.00001**	waterbird
year_since_1969	0.07240634	3.28E-04	158603.847	220.537286	**<0.00001**	waterbird
(Intercept)	-19.99579	0.36763656	28.6950331	-54.390103	**<0.00001**	waterfowl
year_since_1969	0.07022692	2.44E-04	222496.518	287.645469	**<0.00001**	waterfowl
(Intercept)	-17.230321	1.64156718	6.98113706	-10.496263	**<0.00001**	other
year_since_1969	0.03624844	3.18E-04	212109.507	113.946304	**<0.00001**	other

parameters	Estimate	Std. Error	df	t value	Adjusted *p* value	Breeding biome
(Intercept)	-16.414673	0.26519014	44.0135452	-61.897751	**<0.00001**	Eastern Forest
year_since_1969	0.03742953	1.55E-04	549790.499	242.096253	**<0.00001**	Eastern Forest
(Intercept)	-16.599323	0.37867722	42.4804646	-43.835019	**<0.00001**	Aridlands
year_since_1969	0.02390003	3.03E-04	141321.107	78.9966579	**<0.00001**	Aridlands
(Intercept)	-16.132047	0.22620762	62.0706469	-71.315223	**<0.00001**	Western Forest
year_since_1969	0.02506117	2.26E-04	220347.243	111.090013	**<0.00001**	Western Forest
(Intercept)	-16.388839	0.43012669	29.3603395	-38.102354	**<0.00001**	Boreal Forest
year_since_1969	0.01920441	2.46E-04	160769.955	78.2086415	**<0.00001**	Boreal Forest
(Intercept)	-20.96377	0.20990683	84.5442781	-99.871784	**<0.00001**	Wetland
year_since_1969	0.06884089	1.86E-04	459346.81	369.23315	**<0.00001**	Wetland
(Intercept)	-16.229741	0.61039783	22.2424569	-26.588791	**<0.00001**	Habitat Generalist
year_since_1969	0.03619846	1.76E-04	601151.166	205.384232	**<0.00001**	Habitat Generalist
(Intercept)	-17.702427	0.5741062	7.8736301	-30.83476	**<0.00001**	Arctic Tundra

year_since_1969	0.03702951	0.00152967	1695.1187	24.2075876	**<0.00001**	Arctic Tundra
(Intercept)	-16.674112	0.36419652	35.687542	-45.783282	**<0.00001**	Forest Generalist
year_since_1969	0.04325518	1.36E-04	691288.292	318.294069	**<0.00001**	Forest Generalist
(Intercept)	-21.106998	1.04333342	3.77053432	-20.230347	**<0.00001**	Coasts
year_since_1969	0.09100524	0.00216601	1534.90013	42.0151681	**<0.00001**	Coasts
(Intercept)	-15.325279	0.49943613	24.8763874	-30.685164	**<0.00001**	Grassland
year_since_1969	0.00435323	2.07E-04	416282.393	21.0030585	0.16057	Grassland
(Intercept)	-17.230321	1.64156718	6.98113706	-10.496263	**<0.00001**	Introduced
year_since_1969	0.03624844	3.18E-04	212109.507	113.946304	**<0.00001**	Introduced

Table S2. Statistics for the latitudinal shifts of each predator, estimated from linear models between the latitudinal centroid and time (see *Methods*).

**Estimate**	**SE**	**statistic**	**Predator**	**Adjusted *p* value**
-0.0105081	0.00239671	-4.3843762	*Falco sparverius*	**0.000102**
-0.0380354	0.00763398	-4.9823748	*Buteo swainsoni*	**0.0000145**
0.00801418	0.0028087	2.85333823	*Buteo jamaicensis*	**0.00848**
-0.0254503	0.00153488	-16.581306	*Accipiter cooperii*	**<0.00001**
0.01192226	0.0011822	10.0848379	*Aquila chrysaetos*	**<0.00001**
0.0073343	8.38E-04	8.74936822	*Falco mexicanus*	**<0.00001**
0.02605704	0.00347729	7.49349361	*Buteo regalis*	**<0.00001**
0.02313013	0.00176773	13.0846676	*Buteo lineatus*	**<0.00001**
-0.1334401	0.00419295	-31.824898	*Haliaeetus leucocephalus*	**<0.00001**
-0.0044008	0.00386101	-1.1398061	*Buteo platypterus*	0.291
0.00911015	0.00170047	5.35742392	*Accipiter striatus*	**<0.00001**
0.00884313	0.00114396	7.7302572	*Accipiter atricapillus*	**<0.00001**
0.01299551	0.00287001	4.52803031	*Falco peregrinus*	**0.0000656**
-0.0055129	7.33E-04	-7.5221904	*Falco columbarius*	**<0.00001**

Table S3. Statistics of the linear model with spatial lag between turnover of predator-prey interactions and environmental changes between the years 1970 and 2021. Turnover was calculated as the difference between matrices of interaction potentials, including recorded predator-prey interactions only. Environmental change includes changes in land use, mean annual temperature, and mean annual precipitation, and the interaction between changes in temperature and precipitation within each local community. See *Methods* for details on model fitting. Compare to Table 1's results with both recorded and predicted interactions.

**Environmental change**	**Estimate**	**SE**	***z* value**	***p* value**
(Intercept)	0.71943	0.16849	4.26978	**0.00002**
Temperature	0.03778	0.05962	0.63377	0.52623
Precipitation	0.00287	0.07015	0.04088	0.96739
Settlements	0.35137	0.07956	4.41617	**0.00001**
Cultured	0.08890	0.12783	0.69540	0.48680
Agriculture	0.09862	0.12888	0.76523	0.44414
Temperature: precipitation	0.05110	0.06220	0.82164	0.41128
N = 1179, df = 1170; ρ = 0.899, *p* value for $\rho < 0.0001$; pseudo R^2 = 0.502				

Table S4. Statistics of the linear model with spatial lag between turnover of predator-prey interactions and environmental changes between the years 1970 and 2021. These results are part of the sensitivity analysis, where we allowed for the threshold of functional compatibility to scale with the number of species in a community. Turnover was calculated as the difference between matrices of interaction potentials, including both recorded and predicted predator-prey interactions. Environmental change includes changes in land use, mean annual temperature, and mean annual precipitation, and the interaction between changes in temperature and precipitation within each local community. See *Methods* for details on model fitting. Compare to Table 1, where functional compatibility does not scale with the number of species in a community.

**Environmental change**	**Estimate**	**SE**	***z* value**	***p* value**
(Intercept)	0.98611	0.21760	4.53184	**0.00001**
Temperature	-0.00042	0.06300	-0.00665	0.99470
Precipitation	0.03339	0.07397	0.45133	0.65175
Settlements	0.22719	0.08352	2.72008	**0.00653**
Cultured	-0.09043	0.13464	-0.67164	0.50181
Agriculture	-0.09433	0.13578	-0.69471	0.48724
Temperature: precipitation	0.03651	0.06546	0.55784	0.57695
N = 1178, df = 1169; ρ = 0.880, *p* value for $\rho < 0.0001$; pseudo R^2 = 0.428				

Table S5. Statistics of linear mixed effects models for the temporal trends of predation risks for prey species by groups. These results are part of the sensitivity analysis, where we allowed for the threshold of functional compatibility to scale with the number of species in a community. The upper table shows the results by functional groups, lower table shows the results by breeding biomes. Compare to Table S1, where functional compatibility does not scale with the number of species in a community.

**Parameters**	**Estimate**	**Std. Error**	**df**	**t value**	**Adjusted *p* value**	**Group**
(Intercept)	-15.830915	0.13363817	266.328219	-118.46103	**<0.00001**	landbird
year_since_1969	0.03131754	8.46E-05	2669727.97	370.052871	**<0.00001**	landbird
(Intercept)	-19.424799	0.37105805	12.6800935	-52.349759	**<0.00001**	shorebird
year_since_1969	0.03671585	3.39E-04	88906.4721	108.300823	**<0.00001**	shorebird
(Intercept)	-21.431799	0.24456654	43.9995011	-87.63177	**<0.00001**	waterbird
year_since_1969	0.07271737	3.30E-04	154965.98	220.224228	**<0.00001**	waterbird
(Intercept)	-20.276204	0.3749187	28.6491383	-54.081604	**<0.00001**	waterfowl
year_since_1969	0.07443062	2.51E-04	213656.781	297.059297	**<0.00001**	waterfowl
(Intercept)	-17.350215	1.50982733	7.08618937	-11.491523	**<0.00001**	other
year_since_1969	0.04985819	3.54E-04	219802.117	140.722753	**<0.00001**	other

**parameters**	**Estimate**	**Std. Error**	**df**	**t value**	**Adjusted *p* value**	**Breeding biome**
(Intercept)	-16.510596	0.28612796	40.6077491	-57.703537	**<0.00001**	Eastern Forest
year_since_1969	0.03692613	1.63E-04	482979.327	226.277083	**<0.00001**	Eastern Forest
(Intercept)	-16.573448	0.35487305	43.4531636	-46.702469	**<0.00001**	Aridlands
year_since_1969	0.02406847	3.10E-04	139605.506	77.6627934	**<0.00001**	Aridlands
(Intercept)	-16.079953	0.23786404	59.3206275	-67.601446	**<0.00001**	Western Forest
year_since_1969	0.0249044	2.29E-04	214624.291	108.571773	**<0.00001**	Western Forest
(Intercept)	-16.496221	0.42703138	29.535713	-38.629998	**<0.00001**	Boreal Forest
year_since_1969	0.02055381	2.60E-04	144129.764	78.9239838	**<0.00001**	Boreal Forest
(Intercept)	-21.149892	0.19029974	88.2949882	-111.13989	**<0.00001**	Wetland
year_since_1969	0.07146027	1.90E-04	437624.317	376.781697	**<0.00001**	Wetland

(Intercept)	-16.224505	0.60481837	22.0208031	-26.825417	**<0.00001**	Habitat Generalist
year_since_1969	0.03609136	1.77E-04	601827.075	203.382656	**<0.00001**	Habitat Generalist
(Intercept)	-17.70426	0.57372769	7.88674417	-30.858298	**<0.00001**	Arctic Tundra
year_since_1969	0.03702689	0.00152971	1695.11876	24.2051941	**<0.00001**	Arctic Tundra
(Intercept)	-16.625161	0.36579604	35.573756	-45.449266	**<0.00001**	Forest Generalist
year_since_1969	0.04268586	1.37E-04	686870.308	312.095903	**<0.00001**	Forest Generalist
(Intercept)	-21.106998	1.04333342	3.77053432	-20.230347	**<0.00001**	Coasts
year_since_1969	0.09100524	0.00216601	1534.90013	42.0151681	**<0.00001**	Coasts
(Intercept)	-15.408926	0.49326552	24.9097584	-31.238603	**<0.00001**	Grassland
year_since_1969	0.00477542	2.09E-04	412902.548	22.8267523	0.16057	Grassland
(Intercept)	-17.350215	1.50982733	7.08618937	-11.491523	**<0.00001**	Introduced
year_since_1969	0.04985819	3.54E-04	219802.117	140.722753	**<0.00001**	Introduced

Table S6. Summary statistics for interaction potentials by predator species in Figure 2. Data included both recorded and predicted predator-prey interactions. Compare to Table 2, where functional compatibility does not scale with the number of species in a community.

**Predator**	Mean of log-transformed temporal trends of interaction potentials	SD of log-transformed temporal trends of interaction potentials	Number of interaction pairs with decreasing temporal trends	Number of interaction pairs with increasing temporal trends
Falco sparverius	-0.0102	0.0324	25	5
Buteo swainsoni	0.0142	0.0260	10	25
Buteo jamaicensis	0.0285	0.0304	10	68
Accipiter cooperii	0.0379	0.0316	9	163
Aquila chrysaetos	0.0166	0.0472	37	77
Falco mexicanus	0.0207	0.0210	8	50
Buteo regalis	0.0142	0.0253	6	21
Buteo lineatus	0.0488	0.0252	0	16
Haliaeetus leucocephalus	0.1089	0.0599	1	156
Buteo platypterus	0.0117	0.0322	14	21
Accipiter striatus	0.0174	0.0250	21	80
Accipiter atricapillus	0.0005	0.0311	35	40
Falco peregrinus	0.0663	0.0164	0	35
Falco columbarius	0.0517	0.0371	3	42

Table S7. Statistics of the linear model with spatial lag between turnover of predator-prey interactions and environmental changes between the years 1970 and 2021. These results are part of the sensitivity analysis, where we calculated interaction potentials using absolute instead of relative abundances. Turnover was calculated as the difference between matrices of interaction potentials, including both recorded and predicted predator-prey interactions. Environmental change includes changes in land use, mean annual temperature, and mean annual precipitation, and the interaction between changes in temperature and precipitation within each local community. See *Methods* for details on model fitting. Compare to Table 1's results with relative abundances.

**Environmental change**	**Estimate**	**SE**	***z* value**	***p* value**
(Intercept)	1.24448	0.29112	4.27484	**0.00002**
Temperature	-0.01817	0.12301	-0.14773	0.88256
Precipitation	-0.00287	0.14317	-0.02004	0.98401
Settlements	0.44601	0.16262	2.74262	**0.00610**
Wild	-0.07161	0.26281	-0.27246	0.78527
Cultured	-0.01518	0.26501	-0.05727	0.95433
Temperature: precipitation	0.12023	0.12800	0.93929	0.34758
N = 1178, df = 1169; ρ = 0.881, *p* value for ρ < 0.0001; pseudo R^2 = 0.423				

Table S8. Statistics of linear mixed effects models for the temporal trends of predation risks for prey species by groups. These results are part of the sensitivity analysis, where we calculated interaction potentials using absolute instead of relative abundances. The upper table shows the results by functional groups, lower table shows the results by breeding biomes. Compare to Table S1's results with relative abundances.

**Parameters**	**Estimate**	**Std. Error**	**df**	**t value**	**Adjusted *p* value**	**Group**
(Intercept)	-3.6927438	0.13090708	262.553382	-28.208892	**<0.00001**	landbird
year_since_1969	0.02252683	8.22E-05	2773345.25	273.970656	**<0.00001**	landbird
(Intercept)	-7.3167315	0.37247959	12.529302	-19.643309	**<0.00001**	shorebird
year_since_1969	0.03010477	3.33E-04	92543.9874	90.3474414	**<0.00001**	shorebird
(Intercept)	-9.2637264	0.24434754	43.8008072	-37.912091	**<0.00001**	waterbird
year_since_1969	0.06315476	3.29E-04	158603.227	192.124098	**<0.00001**	waterbird
(Intercept)	-7.8335009	0.36630453	28.5603329	-21.385214	**<0.00001**	waterfowl
year_since_1969	0.06263147	2.43E-04	222495.701	257.781874	**<0.00001**	waterfowl
(Intercept)	-5.0843109	1.64150944	6.99440884	-3.0973388	**0.01740462**	other
year_since_1969	0.02689909	3.18E-04	212110.091	84.6634812	**<0.00001**	other

**parameters**	**Estimate**	**Std. Error**	**df**	**t value**	**Adjusted *p* value**	**Breeding biome**
(Intercept)	-4.0815016	0.2660158 1	44.577994 8	-15.343079	**<0.00001**	Eastern Forest
year_since_196 9	0.0276104	1.54E-04	549791.06 3	178.77351 2	**<0.00001**	Eastern Forest
(Intercept)	-4.8382436	0.3753721 6	41.037861 4	-12.889191	**<0.00001**	Aridlands
year_since_196 9	0.0157451 1	3.01E-04	141319.89 6	52.255376 2	**<0.00001**	Aridlands
(Intercept)	-4.3700749	0.2253622 5	61.137616 4	-19.391335	**<0.00001**	Western Forest
year_since_196 9	0.0175215 7	2.25E-04	220348.61 4	78.033093	**<0.00001**	Western Forest
(Intercept)	-4.0581056	0.4285177	28.934445 5	-9.4701003	**<0.00001**	Boreal Forest
year_since_196 9	0.0096417 1	2.47E-04	160769.73 8	39.065709 7	**<0.00001**	Boreal Forest
(Intercept)	-8.7844245	0.2088700 7	82.421561 4	-42.056884	**<0.00001**	Wetland
year_since_196 9	0.0604716 1	1.86E-04	459345.64 4	325.14238 6	**<0.00001**	Wetland
(Intercept)	-4.1094569	0.6102221 4	22.190855 2	-6.7343622	**<0.00001**	Habitat Generalist
year_since_196 9	0.0273214 2	1.76E-04	601150.4	155.49106 1	**<0.00001**	Habitat Generalist
(Intercept)	-5.4684316	0.5255163 1	8.5265158 5	-10.405827	**<0.00001**	Arctic Tundra

year_since_1969	0.0295415	0.00143864	1695.10355	20.534344	**<0.00001**	Arctic Tundra
(Intercept)	-4.5001501	0.36473431	35.8256537	-12.338159	**<0.00001**	Forest Generalist
year_since_1969	0.03404694	1.36E-04	691290.255	251.092501	**<0.00001**	Forest Generalist
(Intercept)	-8.6647211	1.10381835	3.45716662	-7.8497708	**0.00254457**	Coasts
year_since_1969	0.06672674	0.00229363	1534.9571	29.0921444	**<0.00001**	Coasts
(Intercept)	-3.2261123	0.4982699	24.6427172	-6.474628	**<0.00001**	Grassland
year_since_1969	-0.0036441	2.07E-04	416279.93	-17.563367	**<0.00001**	Grassland
(Intercept)	-5.0843109	1.64150944	6.99440884	-3.0973388	**0.01740462**	Introduced
year_since_1969	0.02689909	3.18E-04	212110.091	84.6634812	**<0.00001**	Introduced

Table S9. Statistics for the latitudinal shifts of each predator, estimated from linear models between the latitudinal centroid and time (see *Methods*). These results are part of the sensitivity analysis, where we calculated interaction potentials using absolute instead of relative abundances. Compare to Table S2's results with relative abundances.

**Estimate**	**SE**	**statistic**	**Predator**	**Adjusted *p* value**
-0.0105822	0.00279373	-3.7878272	*Falco sparverius*	**0.000626**
-0.0343366	0.0072638	-4.7270902	*Buteo swainsoni*	**0.0000327**
0.00712487	0.00260517	2.73489548	*Buteo jamaicensis*	**0.0113**
-0.0208016	0.00152664	-13.625711	*Accipiter cooperii*	**<0.00001**
0.00887031	0.00113572	7.81028866	*Aquila chrysaetos*	**<0.00001**
0.006317	1.09E-04	57.7929878	*Falco mexicanus*	**<0.00001**
0.02109376	0.00346852	6.08148815	*Buteo regalis*	**<0.00001**
0.03764414	0.00173968	21.6385166	*Buteo lineatus*	**<0.00001**
-0.1005975	0.00389444	-25.831058	*Haliaeetus leucocephalus*	**<0.00001**
-5.61E-04	0.00312569	-0.1793258	*Buteo platypterus*	0.87631146
0.00537065	0.00143116	3.75264852	*Accipiter striatus*	**<0.00001**
-0.0061741	2.75E-04	-22.475159	*Accipiter atricapillus*	**<0.00001**
0.01006475	0.00316157	3.18346358	*Falco peregrinus*	**0.00352**
-0.0071981	7.78E-04	-9.2526394	*Falco columbarius*	**<0.00001**
